# FVB/NJ Mice Are a Useful Model for Examining Cardiac Adaptations to Treadmill Exercise

**DOI:** 10.3389/fphys.2016.00636

**Published:** 2016-12-21

**Authors:** Andrew A. Gibb, Lindsey A. McNally, Daniel W. Riggs, Daniel J. Conklin, Aruni Bhatnagar, Bradford G. Hill

**Affiliations:** ^1^Department of Medicine, Institute of Molecular Cardiology, University of LouisvilleLouisville, KY, USA; ^2^Diabetes and Obesity Center, University of LouisvilleLouisville, KY, USA; ^3^Department of Physiology, University of LouisvilleLouisville, KY, USA; ^4^Department of Biochemistry and Molecular Genetics, University of LouisvilleLouisville, KY, USA

**Keywords:** cardiac hypertrophy, physical activity, exercise, mouse strain, mitochondria, circadian, compliance, metabolism

## Abstract

Mice are commonly used to examine the mechanisms by which exercise improves cardiometabolic health; however, exercise compliance and adaptations are often strain-dependent or are variable due to inconsistency in exercise training protocols. In this study, we examined nocturnal/diurnal behavior, treadmill exercise compliance, and systemic as well as cardiac-specific exercise adaptations in two commonly used mouse strains, C57BL/6J, and FVB/NJ mice. Metabolic cage analysis indicated a strong nocturnal nature of C57BL/6J mice, whereas FVB/NJ mice showed no circadian element to activity, food or water intake, VO_2_, or VCO_2_. Initial exercise capacity tests revealed that, compared with C57BL/6J mice, FVB/NJ mice are capable of achieving nearly 2-fold higher workloads prior to exhaustion. FVB/NJ mice tested during the day were capable of achieving significantly more work compared with their night-tested counterparts. Following 4 weeks of training, FVB/NJ mice showed significant increases in exercise capacity as well as physiologic cardiac growth characterized by enlarged myocytes and higher mitochondrial DNA content. C57BL/6J mice showed no increases in exercise capacity or cardiac growth regardless of whether they exercised during the day or the night. This lack of adaptation in C57BL/6J mice was attributable, at least in part, to their progressive loss of compliance to the treadmill training protocol. We conclude that the FVB/NJ strain is a useful and robust mouse model for examining cardiac adaptations to treadmill exercise and that treadmill training during daytime hours does not negatively affect exercise compliance or capacity.

## Introduction

Regular exercise improves cardiovascular health (Blair et al., 1996; Mora et al., 2007; Joyner and Green, 2009), augments musculoskeletal function (Egan and Zierath, 2013), and increases both healthspan (Mercken et al., 2012; Egan and Zierath, 2013; de Cabo et al., 2014) and lifespan (Paffenbarger et al., 1986; Blair et al., 1989, 1996; Myers et al., 2002). Nevertheless, the molecular mechanisms by which exercise promotes health are poorly understood (Neufer et al., 2015). Exercise studies commonly use murine models, which are valuable for identifying critical gene programs that contribute to exercise adaptation, primarily, because they offer the benefit of relatively rapid and controlled genetic modification (e.g., Niebauer et al., 1999; Bernstein, 2003; Kemi et al., 2007; Riehle et al., 2014). Nevertheless, sources of variability in such studies could confound our understanding of how exercise mitigates disease or increases overall health. Minimization of confounding factors is an important consideration for designing exercise studies as well as for interpreting the results obtained.

Although mouse models cannot perfectly recapitulate the complex physiological changes occurring in humans with physical activity, they can phenocopy particular aspects of physiological adaptation. For this purpose, three models of mouse exercise are used frequently: treadmill training, forced swimming, and voluntary wheel running. While each of these models have advantages and limitations (Bernstein, 2003; Wang et al., 2010), treadmill training provides the investigative advantage of being able to control the amount of work performed in each training session. This is critical for understanding dependency of (patho) physiological adaptations on exercise workload and becomes increasingly important in studies of mice having different masses (e.g., obesity studies). Unlike the treadmill modality, work cannot be calculated easily in swimming or voluntary wheel exercise. For these reasons, treadmill training is a reliable, well-controlled, and often superior model of exercise for research studies. Nevertheless, several factors contribute to treadmill exercise non-compliance and to exercise-induced adaptations. These include, but are not limited to, mouse strain, exercise environment, acclimatization, motivation, and assessments of exhaustion (Perrino et al., 2011; Platt et al., 2015).

Of these, mouse strain is of principal importance. Inbred strains of mice and rats have pronounced differences in their ability to exercise, or their choice to do so (Ebihara et al., 1978; Barbato et al., 1998; Lightfoot et al., 2001; Lerman et al., 2002; Massett and Berk, 2005). A preponderance of genetic mouse models are on the C57BL/6J or the FVB/NJ background (Taketo et al., 1991; Battey et al., 1999); however, these strains show strikingly different preferences and capacities for exercise. For instance, C57BL/6J mice appear to be poor treadmill runners, yet display superior capacity on voluntary exercise wheels, while the opposite is true for FVB/NJ mice (Lerman et al., 2002; Massett and Berk, 2005). The reason(s) for these differences in training modality preference remain unclear. One reason could relate to the fact that laboratory mice are primarily nocturnal animals, demonstrating the highest activity and food consumption during the night cycle (Kohsaka et al., 2007; Laposky et al., 2008; Arble et al., 2009); however, to our knowledge, the impact of time of exercise (day or night) on treadmill exercise capacity and compliance has not been investigated. The goals of this study were: (1) to test for differences in treadmill exercise capacity and compliance between C57BL/6J and FVB/NJ mice; (2) to delineate whether diurnal or nocturnal training influences exercise compliance and adaptive responses to training; and (3) to assess systemic and cardiac-specific exercise adaptations in mice compliant with treadmill exercise protocols. Our study demonstrates that FVB/NJ mice are a superior strain for treadmill exercise and that the timing of treadmill exercise, at least in this strain, does not influence compliance or exercise-induced adaptation.

## Materials and methods

### Animals

All procedures were approved by the University of Louisville Institutional Animal Care and Use Committee. C57BL/6J and FVB/NJ mice were ordered from Jackson Laboratory (Bay Harbor, ME) at 12 weeks of age and allowed to acclimate at the University of Louisville animal facility for 3 weeks. At 15 weeks of age, male mice were assigned randomly by strain to sedentary (SED) or exercise (EXE-Day or EXE-Night) groups. Food and water were provided *ad libitum*, and the mice were maintained on a 12:12-h light-dark schedule. Because the majority of published studies regarding exercise in mice have utilized male mice, we chose this gender for our study, which enables comparison with the literature. At the conclusion of the study and 24 h after the last exercise session, the mice were fully anesthetized with sodium pentobarbital (40 mg/kg, i.p.), followed by euthanasia via excision of the heart. These procedures are consistent with the AVMA *Guidelines on Euthanasia*.

### Metabolic phenotyping

To assess for differences in basal metabolism and diurnal/nocturnal behavior, metabolic cage analyses were performed in naïve 15-week-old C57BL/6J and FVB/NJ mice, essentially as described (Sansbury et al., 2012; Cummins et al., 2014). Body weight was recorded prior to the initial and final exercise capacity tests to assess for changes in total body mass. Oxygen consumption rates, carbon dioxide production rates, respiratory exchange ratios, food consumption, water consumption and activity (sum of ambulatory and fine movements) were measured using a physiological/metabolic cage system (TSE PhenoMaster System, Bad Homberg, Germany) as described previously (Sansbury et al., 2012; Cummins et al., 2014).

### Exercise capacity testing

**Figure 1:**
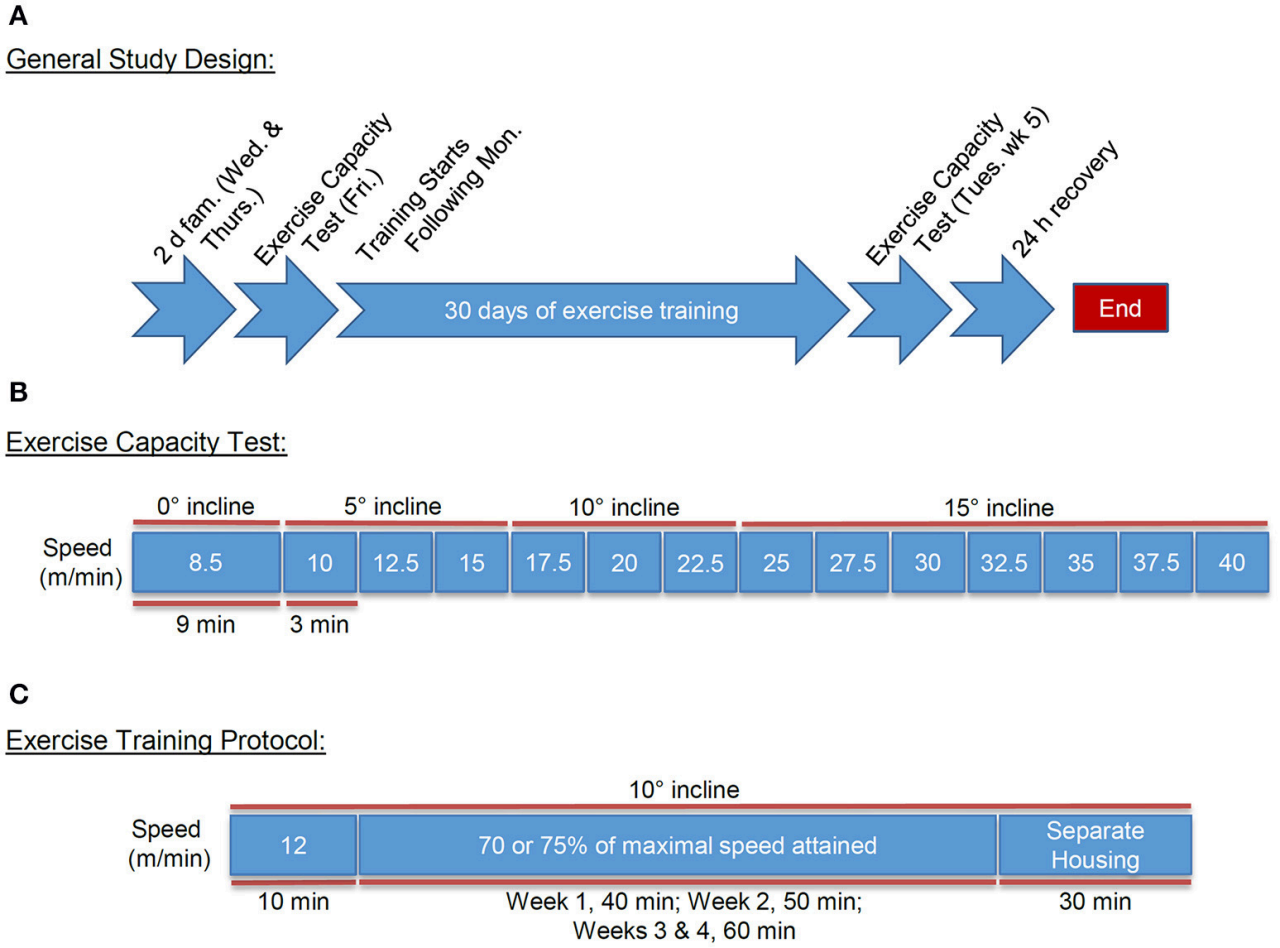
**Exercise testing and training design**. Schematic of the familiarization, testing, and treadmill training design: **(A)** General treadmill training design including: familiarization, pre- and post-exercise capacity testing, and exercise training regimen; **(B)** Exercise capacity testing protocol; and **(C)** Treadmill training regimen including: warm-up and exercise training intensity and durations, and post-training housing.

We performed exercise familiarization and capacity testing in a manner similar to that outlined previously (Massett and Berk, 2005), with minor modifications (Figures 1A,B). Mice were familiarized to the motorized rodent treadmill (Columbus Instruments, Columbus OH) on the Wednesday and Thursday before the first week of training. Familiarization consisted of an initial 10 min period where the treadmill speed and incline were set to zero with shock grid settings of 25 V, 0.34 mA, and 2 Hz. The treadmill speed was then increased steadily to 10 m/min (Wed) and 12 m/min (Thurs) for an additional 10 min.

On the Friday immediately following familiarization to the treadmill, we subjected mice to an exercise capacity test (Figure 1B). For this, the mice were acclimated to the treadmill for 10 min, with the speed and incline set initially to zero. The treadmill speed was then increased to 8.5 m/min with an angle of inclination set to 0° for 9 min. Next, the treadmill speed and incline were increased to 10 m/min and 5°, respectively, for 3 min. The speed was then increased by 2.5 m/min every 3 min to a maximum speed of 40 m/min, while inclination increased by 5° every 9 min until a maximum incline of 15° was achieved.

We developed strict *a priori* criteria for exercise-induced exhaustion. These criteria were: (1) 10 consecutive seconds on the electric grid; (2) spending more than 50% of time on the grid; and/or (3) lack of motivation to manual prodding. Each mouse was removed immediately from their respective lane once one or more of these criteria was reached. Following the protocol, the mice were housed separately for 30 min to avoid noticeable aggressive behavior following exercise.

Following 4 weeks of training, we repeated this testing protocol to assess changes in exercise capacity. Exercise capacity was measured using the parameters of distance run (meters achieved prior to exhaustion) and work accomplished [calculated as the product of body weight (kg) and vertical distance (m); vertical distance = (distance run) (sinθ), where θ = the angle of inclination of the treadmill from 0° to 15°] as outlined previously (Massett and Berk, 2005).

### Exercise training

Mice assigned to exercise training groups were subjected to a 4-week protocol of forced treadmill running. The training protocol commenced the Monday after the initial exercise capacity testing with mice exercising 5 days/week (Mon-Fri) at 70 or 75% of the maximal speed achieved during the initial exercise capacity test and an inclination appropriate to the speed (Figure 1C). Prior to each training bout, we provided mice with a “warm-up” period of 10 min at 0 m/min and 10 min at 12 m/min to promote exercise protocol compliance and to minimize risk of injury. For comparison between the groups, training intensity was set at 20.8 m/min at a 10° incline for FVB/NJ mice and 16.4 m/min at a 5° incline for C57BL/6J mice, which corresponded to 70% of the maximal speed and the appropriate incline at the calculated speed for each strain during the initial exercise capacity test. In subsequent studies of FVB/NJ mice, mice were exercised only during the day, and in these groups, we implemented a more intensive training protocol to further examine systemic and cardiac adaptations to treadmill running. For this, training intensity was set at 75% of the maximal initial exercise capacity, which corresponded to 22.3 m/min at a 10° incline. In all groups, we progressively increased the workload of the mice, such that they trained for 40 min during week 1, 50 min during week 2, and 60 min during weeks 3 and 4. We chose this progressive intensity protocol to prevent training plateau and to stimulate exercise-induced adaptations (Dudley et al., 1982; Hildebrandt et al., 2003; De Angelis et al., 2004; Massett and Berk, 2005).

### Assessment of protocol compliance

To prevent injury and record protocol compliance, we monitored the mice carefully during each exercise session. Upon meeting pre-established indicators of exhaustion, mice were removed from the treadmill, and the time run was recorded (See Supplemental Table 1). We used the percentage of total sessions and total minutes completed throughout the 4-week training program as a measure of compliance. Before and after the initial exercise capacity test, we measured blood lactate levels, which provided a biochemical indicator of exercise-induced exhaustion at or near maximal oxygen consumption (VO_2max_) (McConnell, 1988; Pederson et al., 2005; Ferreira et al., 2007; Hakimi et al., 2007). We recorded lactate concentration in 0.7 μl of blood from a small tail clip (Lactate Plus meter; Nova Biomedical) prior to the protocol and upon meeting the exhaustion criteria defined above. High lactate levels increase confidence in a successful exercise capacity test by ensuring that failure to continue is due to exhaustion at or near VO_2_max and not a failure to comply with the protocol (Von Wittke et al., 1994; Gladden, 2004; Billat et al., 2005).

### Histology

Following euthanasia, tissue was excised and rapidly fixed for immunohistochemical analysis or immediately snap frozen in liquid nitrogen and stored at −80°C. Tissue was fixed in 10% formalin, paraffin embedded, and sectioned at 4 μm. Heart cross-sections were stained with 4′6-diamidino-2-phenylindole (DAPI; Invitrogen) and wheat germ agglutinin (WGA; ThermoFisher) for quantification of cardiomyocyte cross-sectional area. Quantitative measurements were determined using Nikon Elements software.

### Relative mitochondrial DNA measurements

Mitochondrial abundance in heart tissue was estimated by measuring mitochondrial DNA (mtDNA) abundance relative to nuclear DNA (nDNA), similar to our previous studies (Cummins et al., 2014; Salabei et al., 2016). Briefly, total DNA was isolated using a QIAamp DNA Mini Kit (Qiagen). A 25-mg aliquot of the tissue was homogenized, followed by overnight digestion in proteinase K at 55°C. Following isolation, relative amounts of mtDNA and nDNA were compared using quantitative real-time PCR, using 2 ng of the isolated DNA. Primers for cytochrome *b* (mtDNA) and β-actin (nDNA) were used. The sequences are: cytochrome *b*, word5′-TTGGGTTGTTTGATCCTGTTTCG-3′ and 5′-CTTCGCTTTCCACTTCATCTTACC-3′; and β-actin, 5′-CAGGATGCCTCTCTTGCTCT-3′ and 5′-CGTCTTCCCCTCCATCGT-3′.

### Statistical analysis

Unpaired or paired Student's *t*-test was used for direct comparisons; multiple groups were compared by one-way and two-way ANOVA followed by Bonferroni or Sidak Multiple Comparison test, as appropriate. For ratio-based statistical comparisons, the data were log-transformed, and unpaired Student's *t*-test was applied for assessing statistical significance between groups; we used a one-sample *t*-test for intragroup differences from a ratio of 1 (used to determine significant chronobiological differences in metabolic cage endpoints, i.e., food and water intake, physical movement, VO_2_, VCO_2_, RER). A *p* ≤ 0.05 was considered statistically significant.

## Results

### FVB/NJ mice are not nocturnal

**Figure 2:**
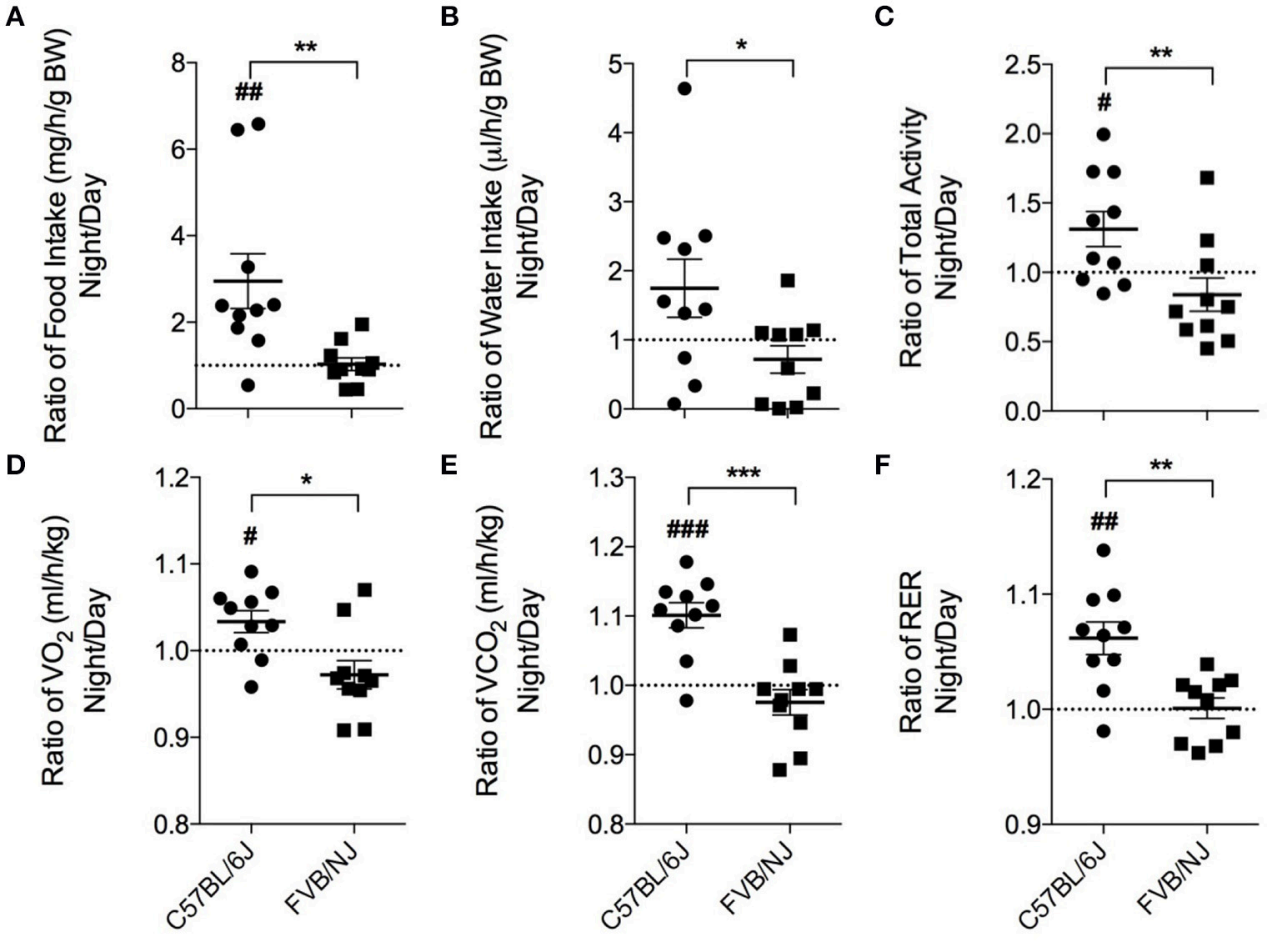
**Chronobiological characteristics of C57BL/6J and FVB/NJ mice**. Ratios of the average night and day values from metabolic cage analysis: **(A)** Food intake, **(B)** Water intake, **(C)** Total activity, **(D)** VO_2_, **(E)** VCO_2_, and **(F)** Respiratory exchange ratio (RER). *n* = 10 per group. ^*^significance between C57BL/6 and FVB/NJ, ^#^significance from a ratio of 1; ^*,#^*p* < 0.05, ^**,*##*^*p* < 0.01, ^***,*###*^*p* < 0.001.

Laboratory mice, in general, are a nocturnal species (Kohsaka et al., 2007; Laposky et al., 2008; Arble et al., 2009), and they typically choose to participate in voluntary exercise at night (Verwey et al., 2013). Thus, the time at which treadmill protocols are executed could be critical for ensuring exercise regimen compliance and adaptation. Therefore, we first examined the circadian characteristics of the mouse strains by measuring their food and water intake, locomotion, VO_2_, VCO_2_ and RER by placing untrained (naïve) mice in metabolic chambers. To assess differences between day and night behavior, a ratio (night values:day values) was calculated for each parameter. Although, overall food consumption was not different between the two strains, the C57BL/6J mice consumed, on average, 3-fold more food at night. In contrast, the FVB/NJ mice showed no difference in food consumption in the night compared with the day (Figure 2A). Cumulative water intake was not different between the strains; however, C57BL/6J mice showed significantly higher water intake at night (Figure 2B). Similarly, C57BL/6J mice were more active at night compared with FVB/NJ mice (Figure 2C). In general, FVB/NJ mice had fewer total beam breaks per hour (C57BL/6J = 2583 ± 711; FVB/NJ = 1736 ± 582; *p* < 0.01), indicating that cage behavioral activity is lower in this strain.

Metabolic analysis yielded analogous results. In C57BL/6J mice, oxygen consumption (VO_2_; Figure 2D), carbon dioxide production (VCO_2_; Figure 2E), and respiratory exchange ratios (RER; Figure 2F) were higher at night compared with the day. Conversely, the FVB/NJ mice did not show metabolic differences in the day vs. the night. Thus, the behavioral and metabolic qualities of C57BL/6J mice are supportive of their known nocturnal nature and are in stark contrast to FVB/NJ mice which demonstrate a relative lack of features characteristic of the nocturnal phenotype. Additionally, FVB/NJ mice had higher rates of oxygen consumption (C57BL/6J = 3713 ± 327 ml/h/kg; FVB/NJ = 4058 ± 346 ml/h/kg; *p* < 0.05) which contributed to lower respiratory exchange ratios (C57BL/6J = 0.93 ± 0.02; FVB/NJ = 0.86 ± 0.02; *p* < 0.0001).

### Effect of strain and time of treadmill running on initial exercise capacity

**Figure 3:**
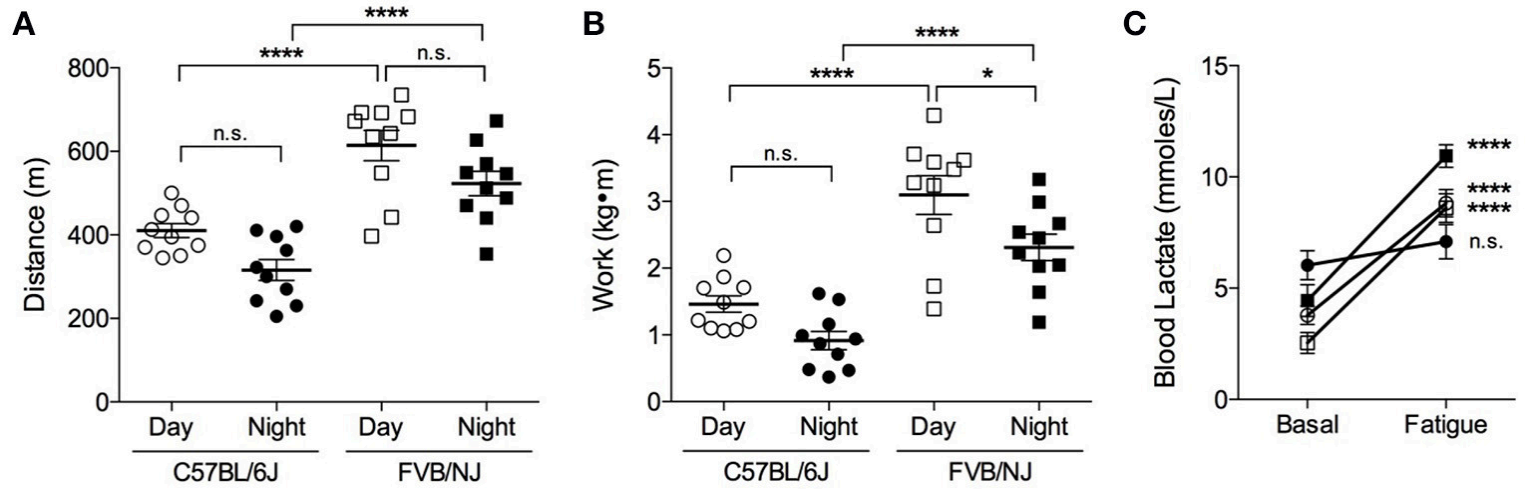
**FVB/NJ mice display a higher initial exercise capacity than C57BL/6J mice**. Measurements of exercise capacity and fatigue in 15-week old C57BL/6J and FVB/NJ mice tested during the day or at night: **(A)** Comparison of distance achieved; **(B)** Comparison of work accomplished; **(C)** Blood lactate levels in C57BL/6J and FVB/NJ mice basally and following fatigue from exercise testing. *n* = 10 mice per group, ^*^p < 0.05, ^****^p < 0.0001.

To determine the effects of mouse genetic background on initial exercise capacity, we subjected 15-week-old C57BL/6J and FVB/NJ mice to exercise capacity testing. The mice were exercised either during the day (i.e., between 9 a.m. and 12 p.m.) under normal laboratory lighting or at night (i.e., between 7:00 p.m. and 10:00 p.m.) under dark room conditions (safelight red lamp). We chose the latter time based on metabolic cage activity data, which showed increased voluntary locomotor activity starting at 6 p.m. Compared with C57BL/6J mice, the FVB/NJ strain ran ~1.5-fold farther (Figure 3A) and demonstrated ~2-fold greater initial capacity for treadmill work (Figure 3B), regardless of when the mice were tested. Despite an apparent trend toward lower initial exercise capacity at night, we found no statistically significant differences between C57BL/6J mice exercised during the day or the night (Figures 3A,B); however, FVB/NJ mice showed significantly lower levels of work performed at night (Figure 3B).

In preliminary assessments, we observed that C57BL/6J mice received more shocks than FVB/NJ mice during treadmill exercise. Therefore, to confirm that reliable exercise capacity values were obtained and to rule out non-compliance to the testing protocol, we recorded blood lactate levels prior to and immediately following the initial exercise capacity test. Resting blood lactate values were similar in C57BL/6J and FVB/NJ mice (Figure 3C). Although, it should be noted that the night C57BL/6J group had a slightly higher resting lactate level, in general, blood lactate increased by 2–4-fold in both strains upon meeting criteria for exhaustion (Figure 3C); both resting and maximal lactate abundances are within the ranges previously published for resting mice and mice exercising at or near their VO_2max_, respectively (Pederson et al., 2005; Ferreira et al., 2007; Hakimi et al., 2007). Collectively, these results suggest that FVB/NJ mice demonstrate a superior ability to perform treadmill work compared with C57BL/6J mice, and they have higher initial exercise capacities when tested during the day.

### Effect of strain and time of training on exercise compliance and adaptation

**Figure 4:**
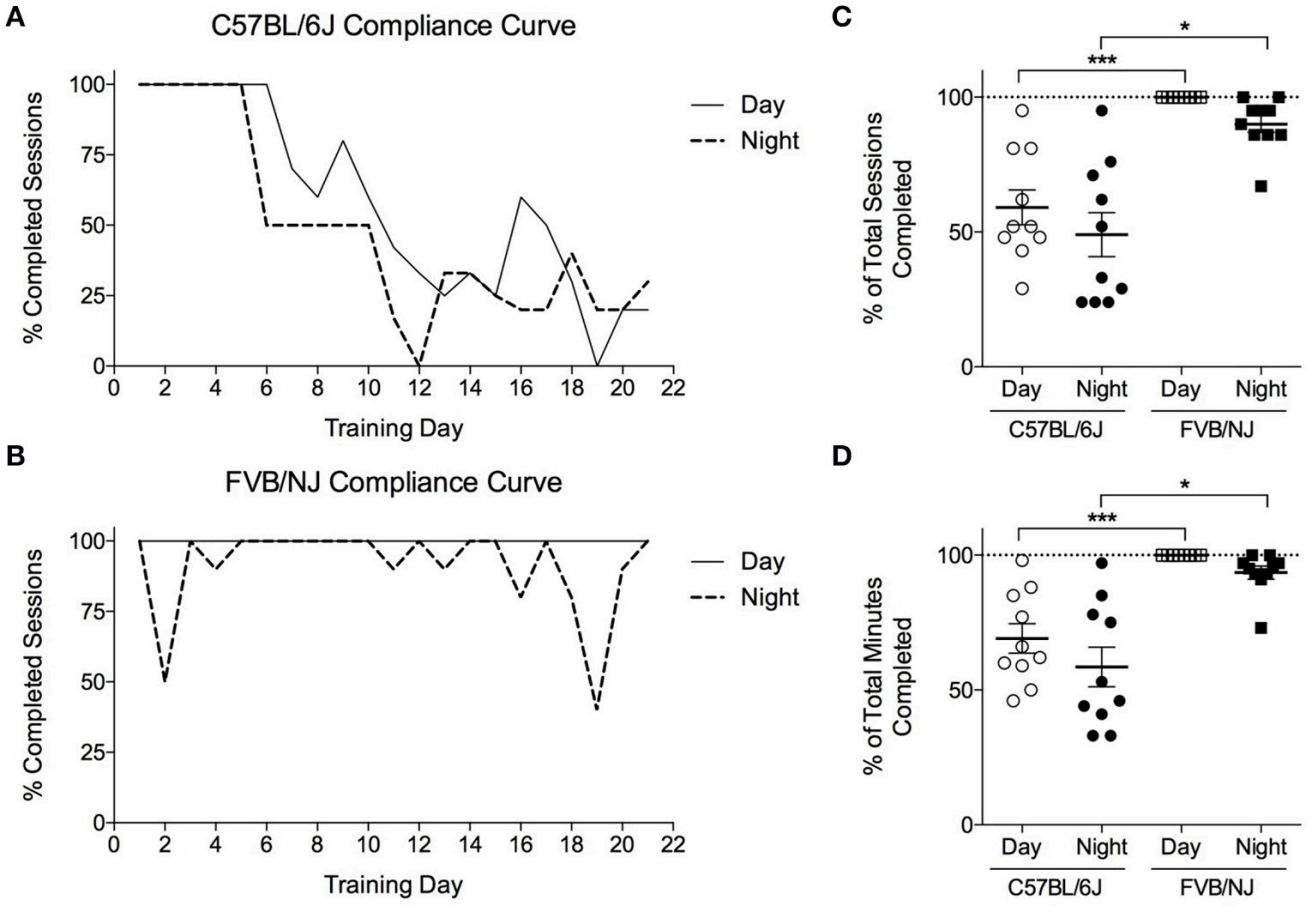
**Compliance of FVB/NJ and C57BL/6J to the treadmill training protocol**. Compliance to a 4-weeks treadmill training protocol in mice: **(A,B)** Compliance curves indicating the percent of mice that completed each training session; **(C)** Compliance measured as the percent of total sessions; or **(D)** Total minutes completed by mice throughout the exercise training program. *n* = 10 per group. ^*^p < 0.05, ^***^p < 0.001.

To determine if mouse strain or the time of training (i.e., day vs. night) influences exercise protocol compliance and adaptive responses to exercise, C57BL/6J and FVB/NJ mice were subjected to a 4-week training program, with one group from each strain training at night, and one group training during the day. Each day, we recorded compliance to the training program for individual mice (Supplemental Table 1). The C57BL/6J mice completed only 50–60% of their training sessions (Figures 4A,C), resulting in less time exercising in general (Figure 4D). Interestingly, compliance in C57BL/6J mice diminished progressively with duration of the training protocol (Figure 4A, Supplemental Table 1). While FVB/NJ mice were generally compliant when exercised during the day or the night, 100% of the day FVB/NJ group complied with the protocol, whereas FVB/NJ mice exercising at night appeared to show modestly compromised compliance (Figures 4B,D); however, this did not achieve statistical significance.

**Figure 5:**
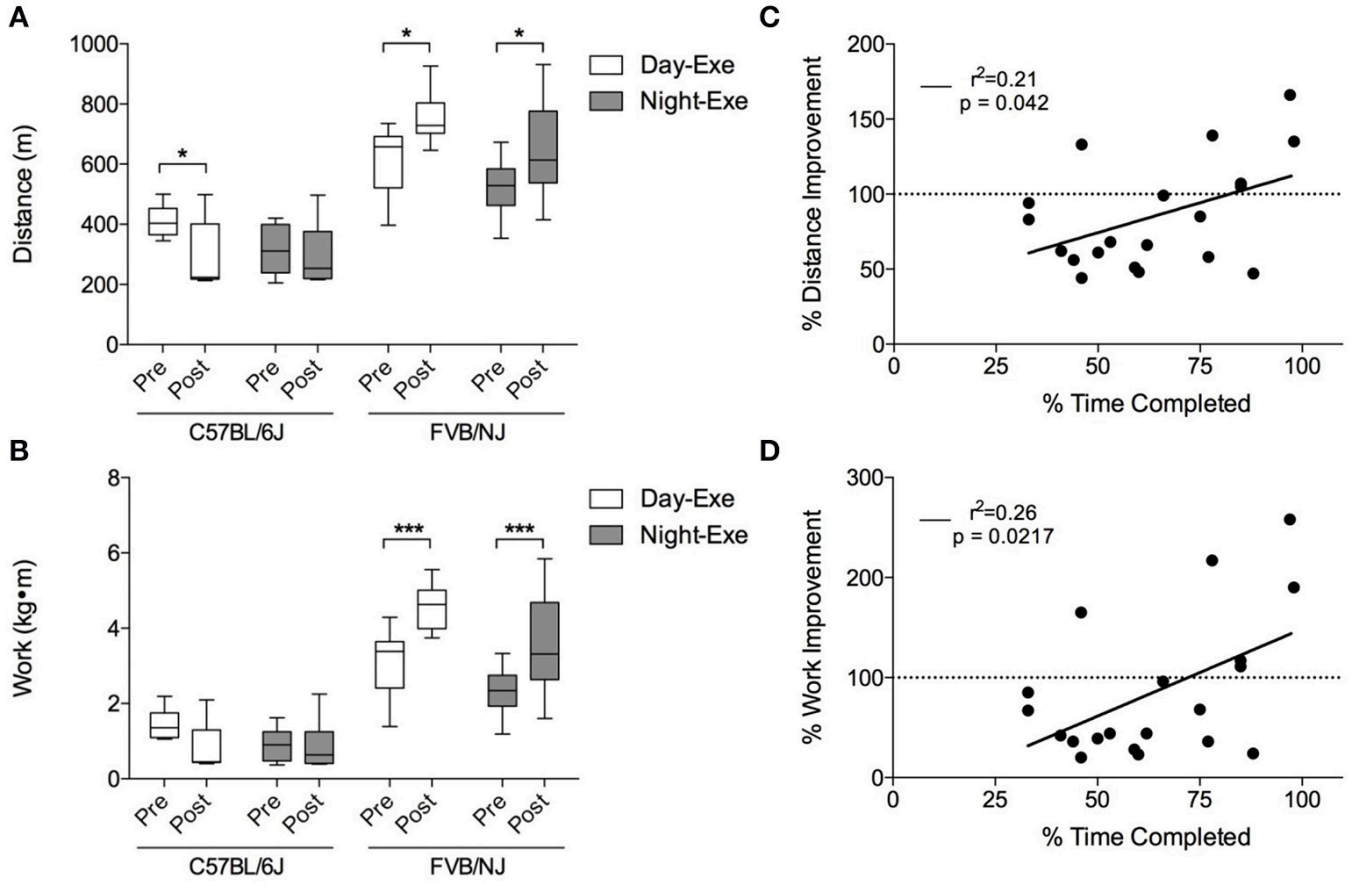
**Treadmill training-induced improvements in exercise capacity in C57BL/6J and FVB/NJ mice**. Changes in exercise capacity in the mouse strains: **(A)** Distance run and **(B)** work accomplished during initial and final exercise testing following 4 weeks of treadmill training. *n* = 10 per group. ^*^p < 0.05, ^***^p < 0.001. Panels C and D show correlations of compliance and **(C)** percent distance and **(D)** percent work improved (in the C57BL/6J strain). *n* = 20 mice (includes the C57BL/6J day and the C57BL/6J night groups).

Pre-training and post-training exercise capacity tests showed that, while both FVB/NJ exercise groups significantly increased exercise capacity after 4 weeks of training, the C57BL/6J mice showed either no improvement or a decrease in the distance run or the work accomplished following training (Figures 5A,B). This lack of response to exercise in the C57BL/6J mice is likely due to poor compliance to the protocol.

To determine if compliance of C57BL/6J mice to the protocol correlated with improvements in exercise capacity, we plotted the percent improvement in distance and work against the percent time completed during the exercise regimen. As shown in Figures 5C,D, there is a significant, albeit weak, correlation between the percent time completed and exercise capacity in this strain. Collectively, these results show that, unlike FVB/NJ mice, C57BL/6J mice are not compliant with this extended treadmill exercise protocol, and that the time at which FVB/NJ mice exercise does not markedly influence their exercise capacity.

Physiological cardiac growth is a common endpoint used to verify cardiometabolic adaptation to exercise (Maillet et al., 2013). Exercise training increased heart weight to tibia length (HW/TL) in both FVB/NJ exercise groups, whereas the C57BL/6J mice showed no changes in heart size (Table 1). We did not find a significant correlation between exercise time completed and HW/TL in the C57BL/6J strain (data not shown). These results indicate that exercise causes cardiac growth in FVB/NJ mice and that C57BL/6J mice, likely due to lack of compliance to this treadmill protocol, fail to demonstrate physiological cardiac growth.

**Table 1:** **Measurements of exercise-induced cardiac growth**.

	**C57BL/6J**	**FVB/NJ**
**BODY MASS, g**		
SED	28.2 ± 2.3	30.5 ± 2.6
EXE-Day	27.3 ± 1.4	29.9 ± 1.7
EXE-Night	26.4 ± 1.8	28.5 ± 1.0
**HEART MASS, mg**		
SED	123.7 ± 15.0	115.4 ± 6.1
EXE-Day	118.7 ± 10.1	135.8 ± 5.5^***^
EXE-Night	118.0 ± 13.0	130.5 ± 9.1^*^
**HW/BW, mg/g**		
SED	4.4 ± 0.4	3.8 ± 0.3
EXE-Day	4.3 ± 0.2	4.6 ± 0.2^****^
EXE-Night	4.5 ± 0.4	4.6 ± 0.2^****^
**HW/TL, mg/mm**		
SED	6.9 ± 0.9	6.3 ± 0.3
EXE-Day	6.6 ± 0.5	7.6 ± 0.3^***^
EXE-Night	6.6 ± 0.7	7.3 ± 0.4^*^

*Mice were trained for 4 weeks at 70% of their initial exercise capacity. Values are means ± SD; n = 10/group. HW/BW, heart weight-to-body weight ratio; HW/TL, heart weight-to-tibia length ratio. Statistical comparisons are between SED and EXE-Day and SED and EXE-Night. n = 10 per group*.

* *p < 0.05*,

*** *p < 0.001*,

**** *p < 0.0001*.

### FVB/NJ mice display robust adaptations following exercise training

**Figure 6:**
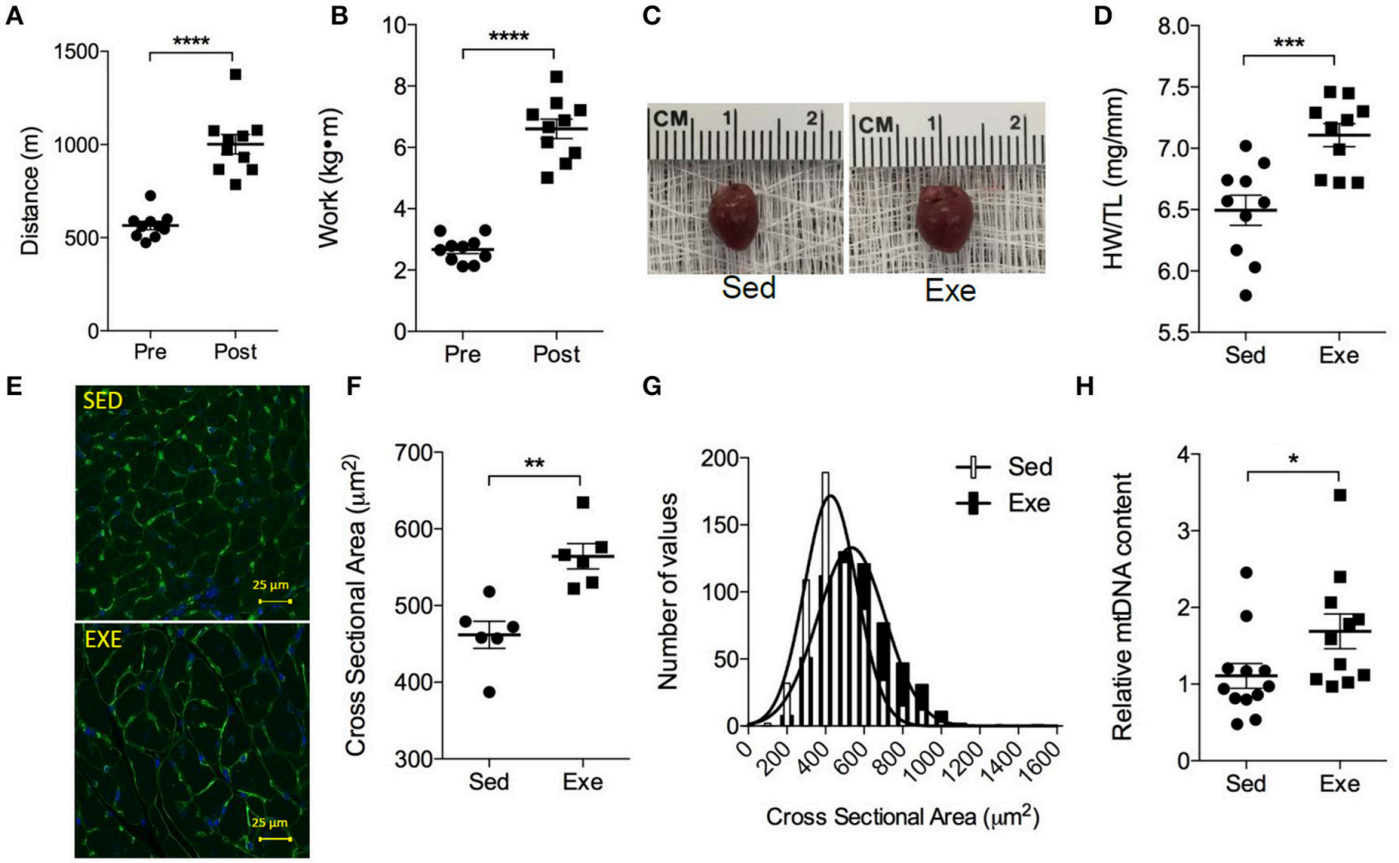
**FVB/NJ mice display robust cardiometabolic adaptations to treadmill exercise training**. Adaptations to a 4-week treadmill training program in FVB/NJ mice, where the training regimen was set based on 75% of their initial exercise capacity: **(A)** Distance run and **(B)** work accomplished during exercise testing; **(C)** Representative images of hearts from Sed and Exe mice; **(D)** HW/TL; **(E)** Representative cross sections of hearts stained with WGA and DAPI; **(F,G)** quantification of myocyte cross sectional area in heart sections; and **(H)** Mitochondrial biogenesis, as indicated by relative mitochondrial (mt) DNA content. *n* = 6–10 mice per group. ^*^p < 0.05, ^**^p < 0.01, ^***^p < 0.001, ^****^p < 0.0001.

It is known that, compared with the C57BL/6J strain, FVB/NJ mice can achieve higher critical running speeds on the treadmill (Billat et al., 2005), which indicates that their training regimen could be intensified further to evoke more robust adaptations to exercise. To test this, we trained an independent group of FVB/NJ mice at a slightly higher intensity (75% of their initial maximum exercise capacity) for 4 weeks. Compared with pre-training exercise capacity values, this more intensive protocol yielded a ~1.7-fold improvement in distance run and a ~2.4-fold increase in work in the mice (Figures 6A,B). As expected, cardiac size was significantly higher in exercised mice (Figures 6C,D). In exercise-adapted hearts, myocyte cross sectional area was found to be 22% higher than that found in hearts of sedentary mice, and the myocyte area distribution curves were shifted to the right (Figures 6E–G), indicating that the exercise-induced cardiac growth in this strain is due primarily to an increase in cardiomyocyte size. Furthermore, mitochondrial biogenesis, estimated using the ratio of relative mtDNA to nDNA content, was 53% higher in the exercise-adapted mice (Figure 6H). Collectively, these results indicate that treadmill exercise in FVB/NJ mice elicits robust systemic and cardiac muscle adaptations.

## Discussion

The goals of this study were to examine treadmill exercise capacity, compliance, and adaptation in two commonly used strains of laboratory mice. We found that C57BL/6J mice have significantly lower treadmill exercise capacity compared with FVB/NJ mice. C57BL/6J mice, in our hands, became progressively non-compliant with the exercise protocol, which is a likely reason underlying their lack of adaptation. Conversely, FVB/NJ mice demonstrated near perfect compliance and showed robust increases in exercise capacity as well as cardiac adaptations. Because laboratory mice are typically nocturnal, we also compared night and day behavior in the strains. Our metabolic cage data suggest that, contrary to the C57BL/6J strain, FVB/NJ mice have no proclivity for activity and feeding at night, and that their metabolic phenotype lacks circadian variation. Moreover, despite the nocturnal nature of C57BL/6J mice, exercising them at night did not improve compliance or exercise capacity. Collectively, these results indicate that FVB/NJ mice are a superior model for examining systemic and tissue-specific adaptation to treadmill exercise and that diurnal training appears to promote higher exercise compliance and capacity.

The increase in exercise capacity and cardiac adaptations in FVB/NJ mice are likely a consequence of their remarkable compliance as well as their superior ability to perform treadmill work. Indeed, other studies show that the FVB/NJ strain has a higher critical running speed (Billat et al., 2005) and is able to run farther than C57BL/6J mice (Lerman et al., 2002; Massett and Berk, 2005). To compare exercise capacity and adaptations between the strains in a controlled manner, we used equivalent relative intensities of training in the strains, which equated to 70% of the speed achieved during initial exercise capacity tests. These experiments showed that FVB/NJ mice could improve their exercise capacity and that exercise in this strain causes physiologic cardiac growth. Training FVB/NJ mice at a higher workload (initiated at 75% of their initial maximum speed) appeared to evoke superior improvements in distance run and work and provoked physiologic cardiac growth. These results indicate that the FVB strain is useful for examining intensity-dependent adaptations to exercise.

The usefulness of the treadmill model lies in its ability to control the level of exercise-associated work and to relate biochemical and physiological adaptations to workload. This is, of course, dependent on compliance to the treadmill protocol. We show that, while C57BL/6J mice completed the protocol during the first week of training, they become increasingly unwilling to run thereafter. It is unlikely that this decrease in compliance is due to overtraining because it occurred upon only the second week of training (and after a 2-day rest period), and the mice did not resume their ability to exercise, even after long periods of refusing to comply with the protocol. Furthermore, the mice did not lose weight during the protocol, which can be an indicator of overtraining (Kadaja et al., 2010). Although, it remains unclear why the C57BL/6J mice choose to run less after the first week, it could be that their tendency to receive more shocks is a negative stimulus for running, or that they associate the shocks with a positive outcome, i.e., removal from the treadmill. Interestingly, C57BL/6J mice are prone to footshock-induced analgesia (Moskowitz et al., 1985; Pavone et al., 1986), and they show progressively decreased shock avoidance behavior compared with other strains (Stavnes and Sprott, 1975). It is also conceivable that this strain is more susceptible to negative stress in the brain caused by forced running (Moraska et al., 2000), as opposed to voluntary running. Thus, it is possible that this strain acclimates to the repeated treadmill shocks that they receive during training.

Escape behavior is another potential contributing factor to the lack of compliance of C57BL/6J mice. A study by Mori and Makino showed that C57BL/6J mice escape and avoid shock by moving to an adjacent compartment in an L-type movement pattern rather than an R-type (rearing and jumping forward) pattern (Mori and Makino, 1994). This is of particular importance because the R-type pattern must be invoked to escape shock in our treadmill apparatus. Regardless, it would appear that the lack of both physiologic cardiac growth and improvements in exercise capacity in C57BL/6J mice is a result of non-compliance with the treadmill protocol.

We also show that FVB/NJ mice lack biological rhythmicity compared with C57BL/6J mice and that the time of day does not appear to affect compliance to the treadmill protocol. Lack of circadian behavior in FVB/NJ mice is a likely consequence of expression of the *retinal degenerative* (*rd*) mutation, which renders them blind to visual images and appears to underlie aberrant circadian wheel running behavior and spatial awareness (Pugh et al., 2004). Conversely, C57BL/6J mice appear to see well: they demonstrate entrainment to a 12:12 h light:dark cycle, and they re-entrain to phase advances (Pugh et al., 2004). Of note, while FVB/NJ mice are blind, C57BL/6J mice become deaf, progressively losing their hearing during the first year of life (Mikaelian, 1979; Henry and Chole, 1980; Willott, 1986). Nevertheless, despite the impaired senses of these strains, the lack of circadian variation in the FVB/NJ strain, and the presence of a nocturnal phenotype in C57BL/6J mice, the timing at which the mice were tested and trained did not appear to have a strong impact. However, we do show that, for FVB/NJ mice, running during the day is associated with a higher initial exercise capacity. Because calculations for the 4-week training regimen are based on this initial capacity, it is possible that testing initial exercise capacities of mice during the day could result in a higher training workload, which could equate to increased physiological adaptations.

Although it is unclear whether the mutations that underlie loss of sight or hearing in FVB/NJ or C57BL/6J mice contribute to their compliance to exercise protocols, it is clear that C57BL/6J and FVB/NJ mice have different exercise capabilities and preferences. Consistent with previous studies (Lerman et al., 2002; Massett and Berk, 2005), we found that C57BL/6J mice have lower treadmill exercise capacity, which did not improve even when they were tested at night. Nevertheless, several studies show that C57BL/6J mice are quite adept at voluntary wheel exercise (e.g., Katzeff et al., 1988; Carter et al., 1995; Lerman et al., 2002; Massett and Berk, 2005; Werner et al., 2008, 2009; Falls et al., 2010; Konhilas et al., 2015), even load bearing wheels which simulate resistance training (Konhilas et al., 2005); and, consistent with their nocturnal behavior, wheel activity in C57BL/6J mice peaks during the night (Pugh et al., 2004). This suggests that the C57BL/6J strain is more amenable to the wheel running modality than treadmill exercise. While several investigators have exercised C57BL/6J mice using a treadmill protocol successfully (reviewed in Perrino et al., 2011), only a few studies document robust increases in exercise capacity or physiological adaptations to extended treadmill training in this strain (Kemi et al., 2002; Ferreira et al., 2010; Sturgeon et al., 2015). Most studies show adaptations that appear relatively minimal compared with other strains (e.g., Massett and Berk, 2005; Ericsson et al., 2010; Miyagi et al., 2014) or demonstrate that additional exercise mimetics (e.g., AMPK or PPARδ agonists) may be required to promote robust physiological adaptations to treadmill exercise in C57BL/6J mice (Narkar et al., 2008).

Several limitations of our study deserve mention. We did not measure VO_2max_ or identify anaerobic thresholds, which can delineate metabolic crossover points (Petrosino et al., 2016); however, we used stringent criteria for determining fatigue (i.e., behavioral criteria decided upon *a priori*) as well as blood lactate measurements to ensure that mice meeting these criteria were fatigued and not simply incompliant with the exercise capacity protocol. Although we show that FVB/NJ mice are proficient treadmill runners, their lack of circadian behavior suggests that they may not be suitable for investigating chronobiological changes associated with exercise (e.g., exercise-induced changes in *clock* genes). In addition, we did not test whether lower speeds in C57BL/6J mice or different motivating stimuli would increase compliance to the protocol. Some studies in rats (Wisløff et al., 2001), as well as C57BL/6J mice (Kemi et al., 2002), use chocolate as a reward-based incentive to comply with the protocol. We did not test the use of reward in our study, nor did we examine whether alternative aversive stimuli (e.g., air puffs instead of shocks) would improve exercise compliance in the C57 strain. Thus, we do not rule out the possibility that C57BL/6J mice could be coaxed to run with an intensity and compliance similar to that of FVB/NJ mice; however, it is clear to us that improved treadmill compliance in C57BL/6J mice would appear to require a different protocol than that used here, or a reward that encourages continual compliance.

In summary, our findings indicate that FVB/NJ mice are a useful strain for testing treadmill exercise-mediated adaptations. This strain complies well with forced treadmill training and shows a robust capacity for cardiac exercise adaptation. Unlike C57BL/6J mice, FVB/NJ mice do not have a strong nocturnal nature, and they appear to show higher initial exercise capacities and comply better when trained during daytime hours. We also found that, in our hands, C57BL/6J mice show poor compliance to the treadmill exercise regimen, regardless of when they exercise. These findings demonstrate that FVB/NJ mice are a suitable and robust model for understanding the mechanisms underlying cardiac adaptations to exercise.

## Author contributions

AG: Design and execution of experiments, analysis of data, manuscript preparation and writing, and financial support; LM: Design and execution of experiments, analysis of data; DR: Statistical analysis; DC: Data analysis and presentation; AB: Experimental design and financial support; and BH: Experimental design, data analysis, manuscript preparation, writing, and financial support.

## Funding

This work was supported in part by grants from the National Institutes of Health [HL122580 (to BH), HL130174 (to BH), GM103492 (to AB)], a Predoctoral Fellowship from the American Heart Association [16PRE31010022 (to AG)], and the American Diabetes Association Pathway to Stop Diabetes Grant [1-16-JDF-041 (to BH)].

### Conflict of interest statement

The authors declare that the research was conducted in the absence of any commercial or financial relationships that could be construed as a potential conflict of interest.

## Supplementary material

Supplementary Table 1 **Exercise compliance chart**.

## References

[B1] Arble D. M. Bass J. Laposky A. D. Vitaterna M. H. Turek F. W. (2009). Circadian timing of food intake contributes to weight gain. Obesity 17, 2100–2102. 10.1038/oby.2009.264 19730426 PMC3499064

[B2] Barbato J. C. Koch L. G. Darvish A. Cicila G. T. Metting P. J. Britton S. L. (1998). Spectrum of aerobic endurance running performance in eleven inbred strains of rats. J. Appl. Physiol. 85, 530–536. 9688730 10.1152/jappl.1998.85.2.530

[B3] Battey J. Jordan E. Cox D. Dove W. (1999). An action plan for mouse genomics. Nat. Genet. 21, 73–75. 10.1038/5012 9916794

[B4] Bernstein D. (2003). Exercise assessment of transgenic models of human cardiovascular disease. Physiol. Genomics 13, 217–226. 10.1152/physiolgenomics.00188.2002 12746466

[B5] Billat V. L. Mouisel E. Roblot N. Melki J. (2005). Inter- and intrastrain variation in mouse critical running speed. J. Appl. Physiol. (1985) 98, 1258–1263. 10.1152/japplphysiol.00991.2004 15542571

[B6] Blair S. N. Kampert J. B. Kohl H. W. III Barlow C. E. Macera C. A. Paffenbarger R. S. Jr.. (1996). Influences of cardiorespiratory fitness and other precursors on cardiovascular disease and all-cause mortality in men and women. JAMA 276, 205–210. 10.1001/jama.1996.03540030039029 8667564

[B7] Blair S. N. Kohl H. W. III Paffenbarger R. S. Jr. Clark D. G. Cooper K. H. Gibbons L. W. (1989). Physical fitness and all-cause mortality. A prospective study of healthy men and women. JAMA 262, 2395–2401. 10.1001/jama.1989.03430170057028 2795824

[B8] Carter G. T. Wineinger M. A. Walsh S. A. Horasek S. J. Abresch R. T. Fowler W. M.. (1995). Effect of voluntary wheel-running exercise on muscles of the mdx mouse. Neuromuscul. Disord. 5, 323–332. 10.1016/0960-8966(94)00063-F 7580246

[B9] Cummins T. D. Holden C. R. Sansbury B. E. Gibb A. A. Shah J. Zafar N.. (2014). Metabolic remodeling of white adipose tissue in obesity. Am. J. Physiol. Endocrinol. Metab. 307, E262–E277. 10.1152/ajpendo.00271.2013 24918202 PMC4121575

[B10] De Angelis K. Wichi R. B. Jesus W. R. Moreira E. D. Morris M. Krieger E. M.. (2004). Exercise training changes autonomic cardiovascular balance in mice. J. Appl. Physiol. 96, 2174–2178. 10.1152/japplphysiol.00870.2003 14729725

[B11] de Cabo R. Carmona-Gutierrez D. Bernier M. Hall M. N. Madeo F. (2014). The search for antiaging interventions: from elixirs to fasting regimens. Cell 157, 1515–1526. 10.1016/j.cell.2014.05.031 24949965 PMC4254402

[B12] Dudley G. A. Abraham W. M. Terjung R. L. (1982). Influence of exercise intensity and duration on biochemical adaptations in skeletal muscle. J. Appl. Physiol. Respir. Environ. Exerc. Physiol. 53, 844–850. 6295989 10.1152/jappl.1982.53.4.844

[B13] Ebihara S. Tsuji K. Kondo K. (1978). Strain differences of the mouse's free-running circadian rhythm in continuous darkness. Physiol. Behav. 20, 795–799. 10.1016/0031-9384(78)90308-6 684115

[B14] Egan B. Zierath J. R. (2013). Exercise metabolism and the molecular regulation of skeletal muscle adaptation. Cell Metab. 17, 162–184. 10.1016/j.cmet.2012.12.012 23395166

[B15] Ericsson M. Andersson K. B. Amundsen B. H. Torp S. H. Sjaastad I. Christensen G.. (2010). High-intensity exercise training in mice with cardiomyocyte-specific disruption of Serca2. J. Appl. Physiol. (1985) 108, 1311–1320. 10.1152/japplphysiol.01133.2009 20167673

[B16] Falls W. A. Fox J. H. MacAulay C. M. (2010). Voluntary exercise improves both learning and consolidation of cued conditioned fear in C57 mice. Behav. Brain Res. 207, 321–331. 10.1016/j.bbr.2009.10.016 19837115

[B17] Ferreira J. C. Bacurau A. V. Bueno C. R. Jr. Cunha T. C. Tanaka L. Y. Jardim M. A.. (2010). Aerobic exercise training improves Ca2^+^ handling and redox status of skeletal muscle in mice. Exp. Biol. Med. (Maywood). 235, 497–505. 10.1258/ebm.2009.009165 20407082

[B18] Ferreira J. C. Rolim N. P. Bartholomeu J. B. Gobatto C. A. Kokubun E. Brum P. C. (2007). Maximal lactate steady state in running mice: effect of exercise training. Clin. Exp. Pharmacol. Physiol. 34, 760–765. 10.1111/j.1440-1681.2007.04635.x 17600553

[B19] Gladden L. B. (2004). Lactate metabolism: a new paradigm for the third millennium. J. Physiol. 558(Pt 1), 5–30. 10.1113/jphysiol.2003.058701 15131240 PMC1664920

[B20] Hakimi P. Yang J. Casadesus G. Massillon D. Tolentino-Silva F. Nye C. K.. (2007). Overexpression of the cytosolic form of phosphoenolpyruvate carboxykinase (GTP) in skeletal muscle repatterns energy metabolism in the mouse. J. Biol. Chem. 282, 32844–32855. 10.1074/jbc.M706127200 17716967 PMC4484620

[B21] Henry K. R. Chole R. A. (1980). Genotypic differences in behavioral, physiological and anatomical expressions of age-related hearing loss in the laboratory mouse. Audiology 19, 369–383. 10.3109/00206098009070071 7436856

[B22] Hildebrandt A. L. Pilegaard H. Neufer P. D. (2003). Differential transcriptional activation of select metabolic genes in response to variations in exercise intensity and duration. Am. J. Physiol. Endocrinol. Metab. 285, E1021–E1027. 10.1152/ajpendo.00234.2003 12902322

[B23] Joyner M. J. Green D. J. (2009). Exercise protects the cardiovascular system: effects beyond traditional risk factors. J. Physiol. 587(Pt 23), 5551–5558. 10.1113/jphysiol.2009.179432 19736305 PMC2805367

[B24] Kadaja L. Eimre M. Paju K. Roosimaa M. Põdramägi T. Kaasik P.. (2010). Impaired oxidative phosphorylation in overtrained rat myocardium. Exp. Clin. Cardiol. 15, e116–e127. 21264069 PMC3016071

[B25] Katzeff H. L. Bovbjerg D. Mark D. A. (1988). Exercise regulation of triiodothyronine metabolism. Am. J. Physiol. 255(6 Pt 1), E824–E828. 3202159 10.1152/ajpendo.1988.255.6.E824

[B26] Kemi O. J. Ellingsen O. Ceci M. Grimaldi S. Smith G. L. Condorelli G.. (2007). Aerobic interval training enhances cardiomyocyte contractility and Ca^2+^ cycling by phosphorylation of CaMKII and Thr-17 of phospholamban. J. Mol. Cell. Cardiol. 43, 354–361. 10.1016/j.yjmcc.2007.06.013 17689560 PMC2995493

[B27] Kemi O. J. Loennechen J. P. Wisloff U. Ellingsen Ø. (2002). Intensity-controlled treadmill running in mice: cardiac and skeletal muscle hypertrophy. J. Appl. Physiol. (1985) 93, 1301–1309. 10.1152/japplphysiol.00231.2002 12235029

[B28] Kohsaka A. Laposky A. D. Ramsey K. M. Estrada C. Joshu C. Kobayashi Y.. (2007). High-fat diet disrupts behavioral and molecular circadian rhythms in mice. Cell Metab. 6, 414–421. 10.1016/j.cmet.2007.09.006 17983587

[B29] Konhilas J. P. Chen H. Luczak E. McKee L. A. Regan J. Watson P. A.. (2015). Diet and sex modify exercise and cardiac adaptation in the mouse. Am. J. Physiol. Heart Circ. Physiol. 308, H135–H145. 10.1152/ajpheart.00532.2014 25398983 PMC4338936

[B30] Konhilas J. P. Widegren U. Allen D. L. Paul A. C. Cleary A. Leinwand L. A. (2005). Loaded wheel running and muscle adaptation in the mouse. Am. J. Physiol. Heart Circ. Physiol. 289, H455–H465. 10.1152/ajpheart.00085.2005 15734890

[B31] Laposky A. D. Bass J. Kohsaka A. Turek F. W. (2008). Sleep and circadian rhythms: key components in the regulation of energy metabolism. FEBS Lett. 582, 142–151. 10.1016/j.febslet.2007.06.079 17707819

[B32] Lerman I. Harrison B. C. Freeman K. Hewett T. E. Allen D. L. Robbins J.. (2002). Genetic variability in forced and voluntary endurance exercise performance in seven inbred mouse strains. J. Appl. Physiol. (1985) 92, 2245–2255. 10.1152/japplphysiol.01045.2001 12015333

[B33] Lightfoot J. T. Turner M. J. Debate K. A. Kleeberger S. R. (2001). Interstrain variation in murine aerobic capacity. Med. Sci. Sports Exerc. 33, 2053–2057. 10.1097/00005768-200112000-00012 11740298

[B34] Maillet M. van Berlo J. H. Molkentin J. D. (2013). Molecular basis of physiological heart growth: fundamental concepts and new players. Nat. Rev. Mol. Cell Biol. 14, 38–48. 10.1038/nrm3495 23258295 PMC4416212

[B35] Massett M. P. Berk B. C. (2005). Strain-dependent differences in responses to exercise training in inbred and hybrid mice. Am. J. Physiol. Regul. Integr. Comp. Physiol. 288, R1006–R1013. 10.1152/ajpregu.00476.2004 15618348

[B36] McConnell T. R. (1988). Practical considerations in the testing of VO_2max_ in runners. Sports Med. 5, 57–68. 10.2165/00007256-198805010-00005 3278356

[B37] Mercken E. M. Carboneau B. A. Krzysik-Walker S. M. de Cabo R. (2012). Of mice and men: the benefits of caloric restriction, exercise, and mimetics. Ageing Res. Rev. 11, 390–398. 10.1016/j.arr.2011.11.005 22210414 PMC3356510

[B38] Mikaelian D. O. (1979). Development and degeneration of hearing in the C57/b16 mouse: relation of electrophysiologic responses from the round window and cochlear nucleus to cochlear anatomy and behavioral responses. Laryngoscope 89, 1–15. 10.1288/00005537-197901000-00001 423642

[B39] Miyagi M. Y. Seelaender M. Castoldi A. de Almeida D. C. Bacurau A. V. Andrade-Oliveira V.. (2014). Long-term aerobic exercise protects against cisplatin-induced nephrotoxicity by modulating the expression of IL-6 and HO-1. PLoS ONE 9:e108543. 10.1371/journal.pone.0108543 25272046 PMC4182716

[B40] Mora S. Cook N. Buring J. E. Ridker P. M. Lee I. M. (2007). Physical activity and reduced risk of cardiovascular events: potential mediating mechanisms. Circulation 116, 2110–2118. 10.1161/CIRCULATIONAHA.107.729939 17967770 PMC2117381

[B41] Moraska A. Deak T. Spencer R. L. Roth D. Fleshner M. (2000). Treadmill running produces both positive and negative physiological adaptations in Sprague-Dawley rats. Am. J. Physiol. Regul. Integr. Comp. Physiol. 279, R1321–R1329. 11004000 10.1152/ajpregu.2000.279.4.R1321

[B42] Mori T. Makino J. (1994). [Response types to shock and avoidance learning in inbred strains of mice]. Shinrigaku Kenkyu 65, 295–302. 10.4992/jjpsy.65.295 7861685

[B43] Moskowitz A. S. Terman G. W. Liebeskind J. C. (1985). Stress-induced analgesia in the mouse: strain comparisons. Pain 23, 67–72. 10.1016/0304-3959(85)90231-3 4058929

[B44] Myers J. Prakash M. Froelicher V. Do D. Partington S. Atwood J. E. (2002). Exercise capacity and mortality among men referred for exercise testing. N. Engl. J. Med. 346, 793–801. 10.1056/NEJMoa011858 11893790

[B45] Narkar V. A. Downes M. Yu R. T. Embler E. Wang Y. X. Banayo E.. (2008). AMPK and PPARdelta agonists are exercise mimetics. Cell 134, 405–415. 10.1016/j.cell.2008.06.051 18674809 PMC2706130

[B46] Neufer P. D. Bamman M. M. Muoio D. M. Bouchard C. Cooper D. M. Goodpaster B. H.. (2015). Understanding the cellular and molecular mechanisms of physical activity-induced health benefits. Cell Metab. 22, 4–11. 10.1016/j.cmet.2015.05.011 26073496

[B47] Niebauer J. Maxwell A. J. Lin P. S. Tsao P. S. Kosek J. Bernstein D.. (1999). Impaired aerobic capacity in hypercholesterolemic mice: partial reversal by exercise training. Am. J. Physiol. 276(4 Pt 2), H1346–H1354. 10199861 10.1152/ajpheart.1999.276.4.H1346

[B48] Paffenbarger R. S. Jr. Hyde R. T. Wing A. L. Hsieh C. C. (1986). Physical activity, all-cause mortality, and longevity of college alumni. N. Engl. J. Med. 314, 605–613. 10.1056/NEJM198603063141003 3945246

[B49] Pavone F. Castellano C. Oliverio A. (1986). Strain-dependent effects of shock-induced release of opioids: dissociation between analgesia and behavioral seizures. Brain Res. 366, 326–328. 10.1016/0006-8993(86)91311-9 3697686

[B50] Pederson B. A. Cope C. R. Schroeder J. M. Smith M. W. Irimia J. M. Thurberg B. L.. (2005). Exercise capacity of mice genetically lacking muscle glycogen synthase: in mice, muscle glycogen is not essential for exercise. J. Biol. Chem. 280, 17260–17265. 10.1074/jbc.M410448200 15711014

[B51] Perrino C. Gargiulo G. Pironti G. Franzone A. Scudiero L. De Laurentis M.. (2011). Cardiovascular effects of treadmill exercise in physiological and pathological preclinical settings. Am. J. Physiol. Heart Circ. Physiol. 300, H1983–H1989. 10.1152/ajpheart.00784.2010 21490325

[B52] Petrosino J. M. Heiss V. J. Maurya S. K. Kalyanasundaram A. Periasamy M. LaFountain R. A.. (2016). Graded maximal exercise testing to assess mouse cardio-metabolic phenotypes. PLoS ONE 11:e0148010. 10.1371/journal.pone.0148010 26859763 PMC4747552

[B53] Platt C. Houstis N. Rosenzweig A. (2015). Using exercise to measure and modify cardiac function. Cell Metab. 21, 227–236. 10.1016/j.cmet.2015.01.014 25651177 PMC4317572

[B54] Pugh P. L. Ahmed S. F. Smith M. I. Upton N. Hunter A. J. (2004). A behavioural characterisation of the FVB/N mouse strain. Behav. Brain Res. 155, 283–289. 10.1016/j.bbr.2004.04.021 15364488

[B55] Riehle C. Wende A. R. Zhu Y. Oliveira K. J. Pereira R. O. Jaishy B. P.. (2014). Insulin receptor substrates are essential for the bioenergetic and hypertrophic response of the heart to exercise training. Mol. Cell. Biol. 34, 3450–3460. 10.1128/MCB.00426-14 25002528 PMC4135616

[B56] Salabei J. K. Lorkiewicz P. K. Mehra P. Gibb A. A. Haberzettl P. Hong K. U.. (2016). Type 2 diabetes dysregulates glucose metabolism in cardiac progenitor cells. J. Biol. Chem. 291, 13634–13648. 10.1074/jbc.M116.722496 27151219 PMC4919448

[B57] Sansbury B. E. Cummins T. D. Tang Y. Hellmann J. Holden C. R. Harbeson M. A.. (2012). Overexpression of endothelial nitric oxide synthase prevents diet-induced obesity and regulates adipocyte phenotype. Circ. Res. 111, 1176–1189. 10.1161/CIRCRESAHA.112.266395 22896587 PMC3707504

[B58] Stavnes K. Sprott R. L. (1975). Effects of age and genotype on acquisition of an active avoidance response in mice. Dev. Psychobiol. 8, 437–445. 10.1002/dev.420080508 1225707

[B59] Sturgeon K. Muthukumaran G. Ding D. Bajulaiye A. Ferrari V. Libonati J. R. (2015). Moderate-intensity treadmill exercise training decreases murine cardiomyocyte cross-sectional area. Physiol. Rep. 3:e12406. 10.14814/phy2.12406 25991723 PMC4463834

[B60] Taketo M. Schroeder A. C. Mobraaten L. E. Gunning K. B. Hanten G. Fox R. R.. (1991). FVB/N: an inbred mouse strain preferable for transgenic analyses. Proc. Natl. Acad. Sci. U.S.A. 88, 2065–2069. 10.1073/pnas.88.6.2065 1848692 PMC51169

[B61] Verwey M. Robinson B. Amir S. (2013). Recording and analysis of circadian rhythms in running-wheel activity in rodents. J. Vis. Exp. 71:e50186. 10.3791/50186 23380887 PMC3582575

[B62] Von Wittke P. Lindner A. Deegen E. Sommer H. (1994). Effects of training on blood lactate-running speed relationship in thoroughbred racehorses. J. Appl. Physiol. (1985) 77, 298–302. 7961248 10.1152/jappl.1994.77.1.298

[B63] Wang Y. Wisloff U. Kemi O. J. (2010). Animal models in the study of exercise-induced cardiac hypertrophy. Physiol. Res. 59, 633–644. 20406038 10.33549/physiolres.931928

[B64] Werner C. Fürster T. Widmann T. Pöss J. Roggia C. Hanhoun M.. (2009). Physical exercise prevents cellular senescence in circulating leukocytes and in the vessel wall. Circulation 120, 2438–2447. 10.1161/CIRCULATIONAHA.109.861005 19948976

[B65] Werner C. Hanhoun M. Widmann T. Kazakov A. Semenov A. Pöss J.. (2008). Effects of physical exercise on myocardial telomere-regulating proteins, survival pathways, and apoptosis. J. Am. Coll. Cardiol. 52, 470–482. 10.1016/j.jacc.2008.04.034 18672169

[B66] Willott J. F. (1986). Effects of aging, hearing loss, and anatomical location on thresholds of inferior colliculus neurons in C57BL/6 and CBA mice. J. Neurophysiol. 56, 391–408. 3760927 10.1152/jn.1986.56.2.391

[B67] Wisløff U. Helgerud J. Kemi O. J. Ellingsen O. (2001). Intensity-controlled treadmill running in rats: VO(2 max) and cardiac hypertrophy. Am. J. Physiol. Heart Circ. Physiol. 280, H1301–H1310. 11179077 10.1152/ajpheart.2001.280.3.H1301

