# Corrigendum: FVB/NJ Mice Are a Useful Model for Examining Cardiac Adaptations to Treadmill Exercise

**DOI:** 10.3389/fphys.2017.00800

**Published:** 2017-10-10

**Authors:** Andrew A. Gibb, Lindsey A. McNally, Daniel W. Riggs, Daniel J. Conklin, Aruni Bhatnagar, Bradford G. Hill

**Affiliations:** ^1^Department of Medicine, Institute of Molecular Cardiology, University of Louisville, Louisville, KY, United States; ^2^Diabetes and Obesity Center, University of Louisville, Louisville, KY, United States; ^3^Department of Physiology, University of Louisville, Louisville, KY, United States; ^4^Department of Biochemistry and Molecular Genetics, University of Louisville, Louisville, KY, United States

**Keywords:** cardiac hypertrophy, physical activity, exercise, mouse strain, mitochondria, circadian, compliance, metabolism

In the original article, there was an error. We state in the Materials and Methods that “At the conclusion of the study and 24 h after the last exercise session, the mice were fully anesthetized with sodium pentobarbital (40 mg/kg, i.p.), followed by euthanasia via excision of the heart.” The amount of sodium pentobarbital used was 150 mg/kg not 40 mg/kg. A correction has been made to the Materials and Methods. The second to last sentence of the paragraph in the Animals subsection has been changed to:

“At the conclusion of the study, and 24 h after the last exercise session, the mice were fully anesthetized with sodium pentobarbital (150 mg/kg, i.p.), followed by euthanasia via excision of the heart.” The authors apologize for this error, which does not change the scientific conclusions of the article in any way.

## Conflict of interest statement

The authors declare that the research was conducted in the absence of any commercial or financial relationships that could be construed as a potential conflict of interest.

